# Left ventricular dysfunction in patients with suspected pulmonary arterial hypertension*

**DOI:** 10.1590/S1806-37132014000600004

**Published:** 2014

**Authors:** Francisca Gavilanes, José Leonidas Alves Jr, Caio Fernandes, Luis Felipe Lopes Prada, Carlos Viana Poyares Jardim, Luciana Tamie Kato Morinaga, Bruno Arantes Dias, Susana Hoette, Rogerio Souza

**Affiliations:** Universidade de São Paulo, Faculdade de Medicina, Hospital das Clínicas, São Paulo, Brazil. Department of Pulmonology, Instituto do Coração, Hospital das Clínicas da Faculdade de Medicina da Universidade de São Paulo - InCor/HCFMUSP, Heart Institute/University of São Paulo School of Medicine Hospital das Clínicas - São Paulo, Brazil; Universidade de São Paulo, Faculdade de Medicina, Hospital das Clínicas, São Paulo, Brazil. Pulmonary Hypertension Group, Department of Pulmonology, Instituto do Coração, Hospital das Clínicas da Faculdade de Medicina da Universidade de São Paulo - InCor/HCFMUSP, Heart Institute/University of São Paulo School of Medicine Hospital das Clínicas - São Paulo, Brazil; Universidade de São Paulo, Faculdade de Medicina, Hospital das Clínicas, São Paulo, Brazil. Pulmonary Hypertension Group, Department of Pulmonology, Instituto do Coração, Hospital das Clínicas da Faculdade de Medicina da Universidade de São Paulo - InCor/HCFMUSP, Heart Institute/University of São Paulo School of Medicine Hospital das Clínicas - São Paulo, Brazil; Universidade de São Paulo, Faculdade de Medicina, Hospital das Clínicas, São Paulo, Brazil. Pulmonary Hypertension Group, Department of Pulmonology, Instituto do Coração, Hospital das Clínicas da Faculdade de Medicina da Universidade de São Paulo - InCor/HCFMUSP, Heart Institute/University of São Paulo School of Medicine Hospital das Clínicas - São Paulo, Brazil; Universidade de São Paulo, Faculdade de Medicina, Hospital das Clínicas, São Paulo, Brazil. Pulmonary Hypertension Group, Department of Pulmonology, Instituto do Coração, Hospital das Clínicas da Faculdade de Medicina da Universidade de São Paulo - InCor/HCFMUSP, Heart Institute/University of São Paulo School of Medicine Hospital das Clínicas - São Paulo, Brazil; Universidade de São Paulo, Faculdade de Medicina, Hospital das Clínicas, São Paulo, Brazil. Pulmonary Hypertension Group, Department of Pulmonology, Instituto do Coração, Hospital das Clínicas da Faculdade de Medicina da Universidade de São Paulo - InCor/HCFMUSP, Heart Institute/University of São Paulo School of Medicine Hospital das Clínicas - São Paulo, Brazil; Universidade de São Paulo, Faculdade de Medicina, Hospital das Clínicas, São Paulo, Brazil. Pulmonary Hypertension Group, Department of Pulmonology, Instituto do Coração, Hospital das Clínicas da Faculdade de Medicina da Universidade de São Paulo - InCor/HCFMUSP, Heart Institute/University of São Paulo School of Medicine Hospital das Clínicas - São Paulo, Brazil; Universidade de São Paulo, Faculdade de Medicina, Hospital das Clínicas, São Paulo, Brazil. Pulmonary Hypertension Group, Department of Pulmonology, Instituto do Coração, Hospital das Clínicas da Faculdade de Medicina da Universidade de São Paulo - InCor/HCFMUSP, Heart Institute/University of São Paulo School of Medicine Hospital das Clínicas - São Paulo, Brazil; Universidade de São Paulo, Faculdade de Medicina, Hospital das Clínicas, São Paulo, Brazil. Pulmonary Hypertension Group, Department of Pulmonology, Instituto do Coração, Hospital das Clínicas da Faculdade de Medicina da Universidade de São Paulo - InCor/HCFMUSP, Heart Institute/University of São Paulo School of Medicine Hospital das Clínicas - São Paulo, Brazil

**Keywords:** Hypertension, pulmonary, Cardiac catheterization, Ventricular dysfunction, left

## Abstract

**OBJECTIVE:**

To evaluate the role of right heart catheterization in the diagnosis of pulmonary arterial hypertension (PAH).

**METHODS:**

We evaluated clinical, functional, and hemodynamic data from all patients who underwent right heart catheterization because of diagnostic suspicion of PAH-in the absence of severe left ventricular dysfunction (LVD), significant changes in pulmonary function tests, and ventilation/perfusion lung scintigraphy findings consistent with chronic pulmonary thromboembolism-between 2008 and 2013 at our facility.

**RESULTS:**

During the study period, 384 patients underwent diagnostic cardiac catheterization at our facility. Pulmonary hypertension (PH) was confirmed in 302 patients (78.6%). The mean age of those patients was 48.7 years. The patients without PH showed better hemodynamic profiles and lower levels of B-type natriuretic peptide. Nevertheless, 13.8% of the patients without PH were categorized as New York Heart Association functional class III or IV. Of the 218 patients who met the inclusion criteria, 40 (18.3%) and 178 (81.7%) were diagnosed with PH associated with LVD (PH-LVD) and with PAH, respectively. The patients in the HP-LVD group were significantly older than were those in the PAH group (p < 0.0001).

**CONCLUSIONS:**

The proportional difference between the PAH and PH-LVD groups was quite significant, considering the absence of echocardiographic signs suggestive of severe LVD during the pre-catheterization investigation. Our results highlight the fundamental role of cardiac catheterization in the diagnosis of PAH, especially in older patients, in whom the prevalence of LVD that has gone undiagnosed by non-invasive tests is particularly relevant.

## Abstract (pt)

**OBJETIVO:**

Avaliar o papel do cateterismo de câmaras cardíacas direitas no diagnóstico de hipertensão arterial pulmonar (HAP).

**MÉTODOS:**

Entre 2008 e 2013, foram avaliadas as características clínicas, funcionais e hemodinâmicas de todos os pacientes que realizaram cateterismo cardíaco direito por suspeita de HAP em nosso laboratório, depois de afastada a presença de disfunção ventricular esquerda (DVE) grave, de alterações significativas nos testes de função pulmonar ou de resultados de cintilografia pulmonar de inalação/perfusão compatíveis com tromboembolismo pulmonar crônico.

**RESULTADOS:**

Durante o período de estudo, 384 pacientes foram submetidos a cateterismo cardíaco diagnóstico. A hipertensão pulmonar (HP) foi confirmada em 302 pacientes (78,6%). A média de idade desses pacientes foi de 48,7 anos. Os pacientes sem HP apresentaram melhor perfil hemodinâmico e menores níveis de peptídio natriurético do tipo B que aqueles diagnosticados com HP. No entanto, 13,8% dos pacientes sem HP apresentavam-se em classe funcional III/IV do *New York Heart Association*. Dos 218 pacientes que cumpriam os critérios de inclusão, 40 (18,3%) e 178 (81,7%) foram diagnosticados como portadores de HP associada à DVE (HP-DVE) e HAP, respectivamente. O grupo HP-DVE tinha idade significativamente mais avançada que aqueles com HAP (p < 0,0001).

**CONCLUSÕES:**

A diferença proporcional entre os grupos HAP e HP-DVE foi bastante significativa, considerando a inexistência de sinais ecocardiográficos sugestivos de DVE importante como parte da investigação que antecedeu o cateterismo. Nossos resultados reforçam o papel fundamental do cateterismo cardíaco no diagnóstico da HAP, ainda mais em faixas etárias mais avançadas, nas quais a prevalência de DVE não diagnosticada através de exames não invasivos é particularmente importante.

## Introduction

Pulmonary hypertension (PH) is a disease defined by the presence of resting mean pulmonary artery pressure (mPAP) ≥ 25 mmHg, assessed noninvasively.^(^ 1 ^)^ Although this clinical and hemodynamic condition is objective, it may be a consequence of numerous changes in the cardiorespiratory system. These changes may be the primary cause of increased pulmonary circulation pressure levels, primary changes in the pulmonary vessels, left heart dysfunction, changes in the ventilation/perfusion ratio, pulmonary thromboembolism, inflammatory changes in the vascular walls, etc.^(^ 2 ^,^ 3 ^)^ Each of these pathophysiological mechanisms ultimately leads to a particular clinical situation that requires a different therapeutic approach.^(^ 4 ^)^ Therefore, the diagnosis and classification of PH are key steps for the proper management of this disease. Current guidelines recommend a classification based on the invasive identification of the predominant vascular component/mechanism, as well as on the underlying clinical condition, which may be associated with the development of PH. Five groups are thus defined-group 1: pulmonary arterial hypertension (PAH); group 2: PH resulting from left heart disease; group 3: PH resulting from significant hypoxia or lung parenchymal disease; group 4: vascular disease due to chronic thromboembolic disease; and group 5: PH resulting from multifactorial and/or poorly understood mechanisms.^(^ 5 ^)^

In recent decades, PAH has received special attention because of the development of specific medications (prostanoids, phosphodiesterase V inhibitors, and endothelin receptor antagonists),^(^ 6 ^)^ the use of which has resulted in improvements in hemodynamics, functional capacity, quality of life, and survival.^(^ 7 ^-^ 11 ^)^ However, this advance found in group 1 (HAP) has no parallel in group 2 (PH related to left heart disease, which is perhaps the most prevalent of all forms of PH).^(^ 12 ^,^ 13 ^)^ To date, there is no evidence to support the use of the medications developed for the treatment of PAH in patients with PH associated with left ventricular dysfunction (LVD); on the contrary, some studies suggest that such medications have deleterious effects, therefore highlighting how important it is to differentiate between group 1 and group 2 patients properly.^(^ 14 ^)^

When the diagnosis of PH is suspected, the initial aim is to identify the potential causes of the increased pulmonary circulation pressure with noninvasive tests. Therefore, before the definitive diagnosis of PH, the patient undergoes a clinical, laboratory, and radiological assessment that, in theory, can rule out the major cardiac causes of LVD (via echocardiography), parenchymal lung diseases (via chest CT, pulmonary function testing, and ergospirometry), or chronic thromboembolic disease (via ventilation/perfusion scintigraphy and chest CT angiography) as potential etiologies of PH.^(^ 1 ^)^ At the end of this extensive investigation, when the results of the investigation are negative, there is a population carefully screened for the diagnosis of PAH, given that no pathophysiological mechanism other than pulmonary artery involvement alone was identified. Nevertheless, it is necessary to perform right heart catheterization to confirm the presence of PH and characterize the hemodynamic profile of the patients.^(^ 15 ^)^

The objective of the present study was to evaluate the role of right heart catheterization in the diagnosis of PAH, in a population carefully screened with noninvasive tests.

## Methods

All patients referred to the Pulmonary Circulation Group with suspected PH who underwent right heart catheterization in the Laboratory of Hemodynamics of the Heart Institute of the University of São Paulo School of Medicine *Hospital das Clínicas*, located in the city of São Paulo, Brazil, between 2008 and 2013, were included in the study. Patients with severe LVD (left ventricular ejection fraction < 40%) were excluded from the analysis, as were those with significant changes in pulmonary function tests (TLC < 50% of predicted or FEV_1_ < 30% of predicted) and those with ventilation/perfusion lung scintigraphy findings clearly consistent with chronic pulmonary thromboembolism. These criteria were intended to rule out clinical conditions that would clearly explain the presence of significant PH. However, in patients who showed less significant changes in the initial tests, the diagnosis was established only after analysis of invasive hemodynamic measurements in relation to the changes in noninvasive tests. In the presence of proportionality between hemodynamic findings and ventilatory changes, the diagnosis of PH resulting from significant hypoxia or lung parenchymal disease was established.^(^ 16 ^)^ At our facility, the diagnosis of chronic pulmonary thromboembolism is often established in parallel with invasive hemodynamic assessment, which is why those cases were included in the total number of cases but do not represent the total number of patients with this clinical condition who were evaluated in the period.

Right heart catheterization consisted of puncture of the right internal jugular vein, passage of a pulmonary artery catheter under fluoroscopic guidance and under the guidance of right heart and pulmonary artery pressure curves, calculation of cardiac output by thermodilution or by the Fick method, and collection of arterial and mixed venous blood gases, as described elsewhere.^(^ 15 ^)^ All data were acquired digitally; however, for the determination of end-expiratory pulmonary artery occlusion pressure (PAOP) adjustment was manual. The diastolic pulmonary gradient was calculated as the difference between diastolic pulmonary artery pressure and PAOP. In addition, blood samples were collected for determination of serum B-type natriuretic peptide (BNP) levels. The diagnosis of PH was established in accordance with the guidelines of the 5th World Symposium, held in 2013-group 1: PAH is diagnosed if mPAP ≥ 25 mmHg and PAOP ≤ 15 mmHg; and group 2: PH associated with LVD is diagnosed if mPAP ≥ 25 mmHg and PAOP > 15 mmHg.^(^ 5 ^,^ 17 ^)^ In cases in which it was not possible to measure PAOP, we used values of left ventricular end-diastolic pressure, measured by puncturing the right femoral artery and introducing a catheter into the left ventricle.

For statistical analysis, continuous data are expressed as mean and standard deviation, whereas categorical data are expressed as proportion. Sample normality was tested by the Kolmogorov-Smirnov test; data on BNP levels showed non-normal distribution and were log-transformed for analysis. The groups were compared by the unpaired Student's t-test or Fisher's exact test, as appropriate. Values of p < 0.05 were considered statistically significant.

## Results

**Figure 1 -:**
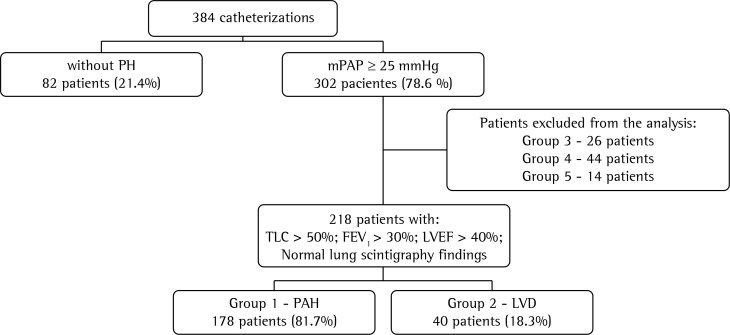
Patient evaluation flowchart. PH: pulmonary hypertension; mPAP: mean pulmonary artery pressure; LVEF: left ventricular ejection fraction; PAH: pulmonary arterial hypertension; and LVD: left ventricular dysfunction.

A total of 384 diagnostic cardiac catheterizations were performed during the study observation period (Figure 1). In 302 patients (78.6%), the presence of PH was confirmed. The patients ranged in age from 19 to 81 years, with a mean age of 48.7 years and a female-to-male ratio of 3.3:1.0.

Despite the echocardiographic finding of increased right ventricular systolic pressure, 21.4% of the patients did not have PH; those patients showed better hemodynamic profiles and lower levels of BNP. However, 13.8% of those patients were categorized as functional class III or IV (Table 1).

**Table 1 -:** Clinical and hemodynamic characteristics among patients diagnosed with pulmonary hypertension and among patients without pulmonary hypertension.a

Characteristics	Groups		p
	Without PH	With PH	
	(n = 82)	(n = 218)	
Age, years	50.3 ± 14.8	48.7 ± 16.0	0.195
Gender, F/M	3.5/1.0	3.3/1.0	1.0
BMI, kg/m^2^	24.8 ± 4.7	26.3 ± 5.6	0.03
FC III/IV, % of patients	13.8	50.0	< 0.001
Hemodynamics			
mPAP, mmHg	18.0 ± 3.3	49.7 ± 18.2	< 0.001
RAP, mmHg	8.0 ± 4.0	11.21 ± 5.7	< 0.001
PAOP, mmHg	11.0 ± 3.0	13.5 ± 6.6	< 0.001
CO, L/min	5.52 ± 2.08	4.49 ± 1.55	0.118
CI, L · min · m^2^	3.3 ± 1.2	2.6 ± 0.8	< 0.001
PVR, IU	1.57 ± 0.99	9.40 ± 6.20	< 0.001
PAC, mL/mmHg	5.10 ± 3.20	1.50 ± 1.08	< 0.001
BNP, pg/mL	98.0 ± 192.0	229.0 ± 311.9	< 0.001

PH: pulmonary hypertension; BMI: body mass index; FC: New York Heart Association functional class; mPAP: mean pulmonary artery pressure; RAP: right atrial pressure; PAOP: pulmonary artery occlusion pressure; CO: cardiac output; CI: cardiac index; PVR: pulmonary vascular resistance; PAC: pulmonary arterial compliance; and BNP: B-type natriuretic peptide.

a Data expressed as mean ± SD, except where otherwise indicated.

At the end of the investigation, we excluded from among the patients with confirmed PH, 84 patients classified as having chronic thromboembolic pulmonary hypertension, PH associated with pulmonary parenchymal diseases, or PH associated with multifactorial or poorly understood mechanisms.

Of the 218 remaining patients, all of whom had PH with no evidence of significant heart or lung disease, 40 (18.3%) were diagnosed with PH associated with LVD (group 2) and 178 (81.7%) were diagnosed with PAH (group 1). The proportional difference between those two groups was quite significant, considering the absence of echocardiographic signs suggestive of severe LVD during the pre-catheterization investigation.

**Figure 2 -:**
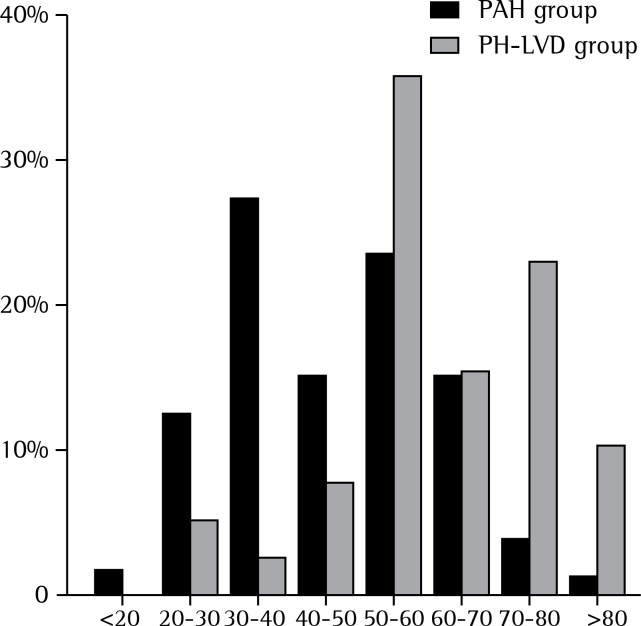
Distribution of pulmonary hypertension diagnosis in the pulmonary arterial hypertension (PAH) group and in the pulmonary hypertension associated with left ventricular dysfunction (PH-LVD) group, by age bracket.

When analyzing the differences found between the two groups, we observed a significant difference regarding age, with group 2 patients being older (p < 0.0001; Table 2). With advancing age, there was an increase in the proportion of patients diagnosed as belonging to group 2 (Figure 2). The clinical presentation of the two groups was similar in terms of functional class, exercise capacity, and level of cardiac output. However, higher levels of BNP, lower levels of mPAP, and, consequently, lower levels of pulmonary vascular resistance, were found in group 2 patients. As expected, group 2 patients showed higher levels of PAOP and lower diastolic pulmonary gradients (Table 2).

**Table 2 -:** Clinical and hemodynamic characteristics among patients diagnosed with pulmonary arterial hypertension and among patients diagnosed with pulmonary hypertension associated with left ventricular dysfunction.a

Characteristics	Groups		p
	PAH	PH-LVD	
	(n = 178)	(n = 40)	
Age, years	46 ± 15	61.2 ± 14.5	< 0.001
Gender, F/M	3.34/1.00	4/1	0.835
BMI, kg/m^2^	26.0 ± 5.5	27.9 ± 5.8	0.06
FC III or IV, % of patients	46.0	47.5	0.86
Hemodynamic			
mPAP, mmHg	53.0 ± 18.0	38.1 ± 12.0	< 0.001
RAP, mmHg	10.4 ± 5.4	14.3 ± 5.9	0.001
PAOP, mmHg	11.3 ± 4.3	22.8 ± 6.6	< 0.001
DPG, mmHg	24.8 ± 14.0	4.1 ± 9.6	< 0.001
CO, L/min	4.4 ± 1.5	4.6 ± 1.7	0.637
CI, L · min · m^2^	2.6 ± 0.7	2.6 ± 0.9	0.88
FC, bpm	81 ± 14	73 ± 10	0.001
PVR, UI	10.49 ± 6.00	4.40 ± 3.85	< 0.001
PAC, mL/mmHg	1.4 ± 0.9	2.3 ± 1.3	0.001
BNP, pg/mL	208 ± 292	345 ± 378	0.004

PAH: pulmonary arterial hypertension; PH-LVD: pulmonary hypertension associated with left ventricular dysfunction; BMI: body mass index; FC: New York Heart Association functional class; mPAP: mean pulmonary artery pressure; RAP: right atrial pressure; PAOP: pulmonary artery occlusion pressure; DPG: diastolic pulmonary gradient; CO: cardiac output; CI: cardiac index; PVR: pulmonary vascular resistance; PAC: pulmonary arterial compliance; and BNP: B-type natriuretic peptide.

a Data expressed as mean ± SD, except where otherwise indicated.

## Discussion

The present study showed that, even in a population carefully screened for the diagnosis of PAH, invasive hemodynamic assessment is fundamental to rule out LVD, hitherto undetected by currently available noninvasive methods. This finding is even more significant in elderly patients, who, despite having clinical characteristics similar to those of group 1 (PAH) patients, have a higher prevalence of PH associated with LVD.

In 21.4% of all patients referred for right heart catheterization because of suspected PH on echocardiography, the diagnosis was not confirmed. This finding is consistent with that reported in a single-center study conducted in the United Kingdom, in which 14% of the patients with suspected PH on echocardiography did not have PH.^(^ 18 ^)^ This proportion, despite being quite significant, may increase further in more specific clinical conditions. In high-output conditions, such as sickle cell anemia, echocardiographic analysis can produce as much as 75% of false-positive results for PH compared with invasive hemodynamic assessment, making the role of right heart catheterization in the diagnostic confirmation of PH even more evident.^(^ 19 ^,^ 20 ^)^ It is also of note that 14% of the patients without PH were categorized as functional class III or IV, which highlights not only the care required in the interpretation of echocardiograms performed during evaluation of dyspnea, but also the need for further evaluation of dyspnea in such patients.

The proportion of patients with a diagnosis of PH associated with LVD that was identified only after right heart catheterization, even after extensive noninvasive investigation, was quite significant (18.3%). This finding highlights the discriminative role of hemodynamic assessment, especially in older patients. In patients over 70 years of age, for instance, the proportion of PH associated with LVD was more than four times as high as that of PAH (Figure 1). This has prognostic and therapeutic implications that are quite relevant.

In addition to the significant difference in age between the patients in the PAH and PH-LVD groups, the latter showed better hemodynamic profiles, although the proportion of patients categorized as functional class III or IV was the same in the two groups. The incident case registry of the United Kingdom, including only patients with PAH, showed that patients over 50 years of age had better hemodynamic profiles, despite having lower functional capacity, characteristics that were attributed to the higher prevalence of comorbidities among older patients.^(^ 18 ^)^

Echocardiographic findings such as left atrial enlargement, abnormal mitral regurgitation, and left ventricular hypertrophy can suggest a diagnosis of heart failure with preserved ejection fraction or other diagnoses, such as constrictive pericarditis and restrictive/infiltrative heart disease, which can lead to the development of PH.^(^ 21 ^)^ However, the absence of this echocardiographic profile does not rule out LVD as the cause of PH, hence the relevance of our findings.

Obtaining a reliable PAOP measurement can be particularly difficult in patients with PH. Not only that, but the time it takes for PAOP to stabilize can vary according to their baseline pathophysiological state^(^ 22 ^)^; therefore, special attention should be paid to the measurement technique so that the best possible curve is obtained.^(^ 23 ^)^ In our study, although it only happened in a few cases, left ventricular end-diastolic pressure was determined when PAOP could not be measured properly, thereby ensuring the accuracy in discriminating between the groups of interest.

Currently, LVD is believed to be the most common cause of PH. Although PH may be present in as much as 25% of the population with heart failure with preserved ejection fraction, among patients with heart failure with reduced left ventricular ejection fraction, as many as two thirds go on to develop PH.^(^ 24 ^)^ Therefore, PH associated with LVD may occur as a result of different clinical conditions that lead to a passive increase in pulmonary artery pressures by retrograde transmission of elevated left atrial pressure. However, it is of note that chronic venous hypertension can also induce pulmonary arterial endothelial dysfunction and abnormal upregulation of neurohormones, cytokines, and other regulators of vascular reactivity, which, under certain circumstances, will lead to vascular remodeling, as in PAH.^(^ 21 ^)^ In such cases, post-capillary PH with a pre-capillary component,^(^ 14 ^)^ which indicates a poorer prognosis,^(^ 25 ^)^ might occur. 

It should be stressed that the fact that our study was conducted in a single referral center is a limitation to the extrapolation of the results. In addition, the initial design did not contemplate collection of data regarding the presence of comorbidities or measurement of other echocardiographic variables, limiting the analysis of other potential markers of LVD. However, the number of new patients diagnosed during the study period makes our findings comparable to those of recently published registries.^(^ 18 ^,^ 26 ^,^ 27 ^)^

In conclusion, in a population suspected of PAH, invasive hemodynamic population is fundamental for correct diagnosis, particularly in advanced age, when the prevalence of LVD increases significantly. In addition, the care required in the interpretation of echocardiographic findings suggestive of PH is of note, given the significant proportion of patients with false-positive results for PH relative to the results of right heart catheterization.
